# Hyperthermia and radiotherapy: physiological basis for a synergistic effect

**DOI:** 10.3389/fonc.2024.1428065

**Published:** 2024-08-06

**Authors:** Michael F. Righini, André Durham, Pelagia G. Tsoutsou

**Affiliations:** ^1^ Faculty of Medicine, University of Geneva (UNIGE), Geneva, Switzerland; ^2^ Department of Radiation Oncology, Geneva University Hospitals (HUG), Geneva, Switzerland

**Keywords:** hyperthermia, radiotherapy, cancer biology, hypoxia, DNA-repair, tumor microenvironment, immunotherapy, thermotolerance

## Abstract

In cancer treatment, mild hyperthermia (HT) represents an old, but recently revived opportunity to increase the efficacy of radiotherapy (RT) without increasing side effects, thereby widening the therapeutic window. HT disrupts cellular homeostasis by acting on multiple targets, and its combination with RT produces synergistic antitumoral effects on specific pathophysiological mechanisms, associated to DNA damage and repair, hypoxia, stemness and immunostimulation. HT is furthermore associated to direct tumor cell kill, particularly in higher temperature levels. A phenomenon of temporary resistance to heat, known as thermotolerance, follows each HT session. Cancer treatment requires innovative concepts and combinations to be tested but, for a meaningful development of clinical trials, the understanding of the underlying mechanisms of the tested modalities is essential. In this mini-review, we aimed to describe the synergistic effects of the combination of HT with RT as well as the phenomena of thermal shock and thermotolerance, in order to stimulate clinicians in new, clinically relevant concepts and combinations, which become particularly relevant in the era of technological advents in both modalities but also cancer immunotherapy.

## Highlights

- The combination of radiotherapy and mild hyperthermia has attracted increasing interest in recent years and remains a promising yet relatively poorly studied approach for cancer treatment. To incite future, innovative concepts of the association of both modalities, their underlying mechanisms, interactions and potential need to be understood. This review summarizes current knowledge of the above and aims to be a helpful resource for guiding new preclinical and clinical studies advancing this opportunity.

## 1 Introduction

Treating cancer with heat is known since the 19^th^ century: W.B. Coley was able to cause their tumor regression, in patients to whom he administered preparations of killed bacterial cultures *(“Coley’s toxin”)*, and thus induced fever (1, 2). Mild hyperthermia (HT) is still today a therapeutic anticancer modality, where a tissue is exogenously heated to temperatures between 39–45°C, using electromagnetic waves, ultrasound, thermal conduction, or hyperthermic infusion (3). Using ionizing irradiation to shrink tumors is known since the early 20^th^ century: since the discovery of radium by Marie Curie, radiotherapy (RT) has made a tremendous progress, both in the fields of biology and technology and became one of the pillars of cancer treatment. The synergistic combination of both HT and RT is known since the 1970s, where thermoradiotherapy (TRT), at first at a preclinical level (2, 4), then confirmed at meta-analysis clinical level, has been shown to improve outcomes, as compared to RT alone, without increasing serious adverse effects, at least for several advanced cancers, such as breast (5), cervical (6), esophageal (7), and head and neck (8). Today, a revived interest in TRT is observed, possibly because the underlying mechanisms of the synergy between RT and HT might create new opportunities to further improve therapeutic outcomes in modern settings, such as, for instance, the context of immunotherapy (9). Therefore, understanding the biological mechanisms of the synergistic effect of TRT might help conceptualize innovative associations to be tested in future clinical studies and becomes clinically relevant. We thus aimed to summarize and put into perspective the established knowledge on the interactions between HT and RT but also their limitations, to probe modern research in the field.

## 2 The synergy of thermoradiotherapy: two pieces in a puzzle

### 2.1 DNA damage

Radiation-induced DNA damage is the pivotal, classical radiobiologic event regarded as the major effect of RT in both tumors and healthy tissues (10). This occurs either by direct ionization or, predominantly, by inducing radiolysis of water molecules, resulting in the production of free-oxygen radicals (ROS), which then induce DNA oxidation (11, 12). Such damage includes DNA-base alterations, DNA-DNA/DNA-protein cross-links, single-strand breaks and double-strand breaks, the latter being the most lethal (13, 14). The accumulation of unrepaired DNA lesions activates the cell-cycle checkpoint machinery (ATM/p53/p21 or ATM/CHK2/CDC25A-C pathways), leading to the temporary arrest of the cell cycle. If DNA damage is not quickly repaired, the cell-cycle checkpoint machinery remains active, thereby promoting cell death (via mitotic catastrophe, apoptosis, senescence or autophagy) (13, 15, 16). Therefore, the greater the efficiency of DNA repair mechanisms, the lower the cytotoxicity of ionizing radiation. This is the key to the therapeutic effect of RT: tumor cells, due to their reduced capacity to repair DNA, are more sensitive to RT than healthy cells, thus creating a therapeutic window that is explored in the clinical setting and makes RT a very efficient anti-cancer treatment (13, 15, 17).

Heat, especially at temperatures exceeding **≈**41°C, damages several proteins involved in DNA repair, called DNA-damage responses (DDRs). DDRs repairing double-strand breaks are particularly affected, i.e., non-homologous end joining (NHEJ), homologous recombination (HR) and back-up non-homologous end joining (back-up NHEJ), thus making HT a great tool to enhance the cytotoxicity of ionizing radiation (14, 15, 18, 19). Moreover, heat also slows DNA replication in tumor cells, thus reducing their reproductive capacity (14, 18, 19). It is highly controversial whether HT, by producing ROS, can *directly* cause single-or double-strand breaks (18, 19).

Radioresistance is dominated by the moment in the cell cycle where exposure to irradiation occurs: cells in the synthesis (S) phase, as compared to cells in G_1_, G_2_ or mitotic (M) phase, are particularly radioresistant, because their DDRs are physiologically upregulated. Therefore, HT, which damages DDRs, can radiosensitize previously radioresistant cells (13, 19). In terms of synergy, both RT and HT effects on DNA damage and repair display a very interesting complementarity, which can widen the therapeutic window of RT.

### 2.2 Hypoxia

Hypoxia is another challenge for therapeutic RT, as it is directly associated to radioresistance. Indeed, as a tumor grows, the local vasculature becomes insufficient to support its growing nutrient needs. Cancer cells and other cells situated in the tumor microenvironment (TME) thus secrete many pro-angiogenic factors, especially vascular endothelial growth factor (VEGF), leading to the development of an abnormal local vascular network. Tumor blood vessels are dilated, tortuous, immature, highly permeable and heterogeneously distributed. This abnormal vascular network is unable to properly vascularize the tumor, tumoral oxygen requirements remain higher than its availability, and the microenvironment becomes hypoxic (20–22).

The generation of ROS by ionizing radiation, the primary mediator of RT cytotoxicity, is chemically dependent on the local partial pressure of oxygen (pO_2_). Therefore, a chronically hypoxic TME favors radioresistance (11, 12, 23). Indeed, hypoxia is associated with poor clinical prognosis, because it not only increases resistance to treatment, but also intrinsically enhances the tumor malignancy, through the activation of hypoxia-inducible factors (HIFs), which stimulate angiogenesis, reduce p53 expression, inhibit apoptosis, and reduce the immune system response (24–27).

HT, up to ≈ 42°C, induces a transient vasodilation of abnormal tumor blood vessels and an increase of vascular permeability, leading to an increase in tumor perfusion. Therefore, the TME regains a normal oxygen partial pressure (pO_2_), a normal concentration of nutrients and a normal pH, which reverse the established radioresistance (14, 25, 28–30). The duration of the reoxygenation is still controversial but may last up to 24 hours (14, 25). Nonetheless, this mechanism becomes counterproductive at temperatures above ≈ 42°C: heat causes vascular damage, leading to vascular occlusion and thus to a prolonged worsening of local hypoxia, with hypoxic cells dying from ischemia (14, 15, 25, 28, 30) Therefore, the radiosensitizing vascular effects of HT may only be achievable at temperatures below ≈ 42°C. Of note, HT-induced reoxygenation of tumor tissue could be further enhanced by the addition of drugs that increase local oxygen availability, such as oxygen mimetics (e.g., misonidazole), mitochondrial respiration inhibitors (e.g., atovaquone), carbogen gas breathing, or oxygen-carrying nanoparticles (11, 28, 31).

### 2.3 Cancer stem cells

Cancer stem cells (CSCs) represent an important challenge of cancer treatment. Indeed, this small subpopulation of cancer cells promotes cancer initiation, progression, metastasis, treatment resistance and recurrence (32–34). They possess some characteristics of normal stem cells, such as self-renewal and unlimited proliferation capacities (14, 31, 33, 35). CSCs are harbored particularly in hypoxic areas of the tumor, because a hypoxic TME promotes stem-like properties, inhibits cellular differentiation and apoptosis (32, 35, 36). These specific microenvironments are called “niches”: they are surrounded and symbiotically collaborate with cancer-associated cells to increase proliferation, resist to treatment, evade the immune system, metastasize and differentiate into multiple tumor cell types (35, 37). Due to oxygen and nutrient local deprivation, a fraction of CSCs, as well as chronically hypoxic tumor cells, is maintained in a state of quiescence (i.e., cell cycle reversibly arrested in G_0_ phase). Quiescent cells no longer undergo mitosis and upregulate their DNA repair pathways, rendering them constitutively resistant to cytotoxic treatments (32, 38) and DNA-damaging agents, such as RT (31–33, 37–39). Some evidences suggests that HT may be able to radiosensitize CSCs, as well as quiescent tumor cells, although the underlying pathophysiological mechanisms are not fully understood, but may include HT-induced TME reoxygenation and damage to DDRs (14, 32, 33, 40, 41).

### 2.4 Immunostimulation

#### 2.4.1 Hyperthermia-induced immunostimulation

HT possesses immunostimulatory effects only. During an infection, the hypothalamus induces fever in response to elevated blood levels of cytokines, especially IL-1, IL-6 and TNF-α. In addition to inhibiting bacterial replication, fever creates a pro-inflammatory state in tissues that allows for increased immunogenicity of immune cells. The immune effects of fever may be reproducible with local HT, potentiating immune targeting of neoplastic cells escaping immunosurveillance (42). Heat-induced activation of the immune system is mediated by heat shock proteins (HSPs); they increase antigen presentation and maturation of dendritic cells, stimulate the phagocytic function of macrophages, induce the release of tumor neoantigens from tumor cells, favor the proliferation and cytotoxicity of CD8+ T and NK cells, promote the activation and proliferation of B lymphocytes and inhibit T-regulatory (Treg) cells. Heat-stimulated macrophages produce cytokines that increase the expression of cell-adhesion molecules on endothelial cells to facilitate leukocyte infiltration into the tissue (42–46). Mild HT also appears to induce immunogenic cell death (ICD), a specific type of apoptosis that promotes an adaptive immune response (44, 45, 47). Finally, the vasodilatory effect and the increase in vascular permeability produced by HT facilitate the arrival and penetration of immune cells into the heated tissue (28, 43).

#### 2.4.2 Radiotherapy-induced immunostimulation/immunosuppression

RT, however, has both immunostimulatory and immunosuppressive effects. Indeed, RT induces local secretion of certain immunostimulatory pro-inflammatory cytokines (IFNs, IL-1β) that facilitate T-cell entry into the tumor (by upregulating adhesion molecules on the endothelium) and induces activation and enhanced antigen-presenting capacity of dendritic cells and T cells (22, 46–48). RT also appears to increase the expression of MHC-I and NKG2D receptors on the tumor surface, which, respectively, facilitates tumor cell recognition by T cells and enhances NK cells activity and cytotoxicity (22, 48–50). RT is thus a known trigger of ICD (22, 48, 50, 51). However, RT induces the secretion of various chemokines, some of which attract immunostimulatory cells (dendritic, T and NK cells), but others immunosuppressing cells (Treg, tumor associated macrophages (TAMs), myeloid-derived suppressor cells (MDSCs)), the latter facilitating tumor progression. Therefore, RT is considered a double-edged sword, in terms of tumor immunogenicity (22, 48). Ionizing radiation also damages leukocytes situated in the tumor and stimulates the secretion of anti-inflammatory cytokines (TGF-β, IL-6, IL-10, CSF-1), which reduce the antigen-presenting abilities of dendritic cells, reduces T CD8+ lymphocytes cytotoxicity and CD4+ cell differentiation, attracts cancer-associated fibroblasts (CAFs), creates a radioresistant state in tumors and contributes to tumor cells’ proliferation and invasion (22, 48). Upregulation of PD-L1 has also been observed after RT (22, 48, 52). Furthermore, doses >10Gy/fraction destroy abnormal fragile tumor vessels: this not only impedes the infiltration of immune cells into the tumor but also exacerbates local hypoxia, thereby inducing tumor radioresistance (22).

Some evidence suggests that the addition of HT to RT leads to an increase in tumor immunogenicity (53–56). However, to optimize the combination of RT-HT from an immunostimulatory point of view, a better understanding of the ideal parameters of both modalities is needed: optimal fractionation, total dose and sequence of RT with immune checkpoint inhibitors (ICIs) are still under investigation, with data suggesting that moderate hypofractionated regimens are the most promising (22, 48–50, 57), while for HT, the ideal temperature frame is unknown, with fever-range temperatures up to 41°C being potential optimal HT parameters for immunostimulation, as some immunostimulatory effects only occur at specific temperatures (42, 43). In the context of RT-induced immunostimulation, a rare, still increasingly sought, phenomenon, called the abscopal effect, has been observed: it consists of the regression of non-irradiated lesions, suggesting a systemic and immunogenic effect of RT, a classically considered “local” treatment. Although incompletely understood, it seems that the abscopal effect represents the distant effect of *in-situ*, tumor-specific immunostimulation, through activation of antigen-presenting cells following the local release of tumor-associated antigens (TAAs) after RT, leading to the activation of T CD8+ lymphocytes and consequent tumor destruction by cell-mediated immunity (22, 48, 49, 57). The abscopal effect seems indeed to occur more frequently when ICIs are administered (48, 49, 57, 58). Interestingly, putative abscopal responses have been reported in patients treated with RT and HT, although these remain at an exploratory level (59), with ferroptosis having been suggested as an implicated mechanism (53–56). Importantly, ICIs can be added to the combination of RT and HT, promising to further enhance the immunostimulatory combination of both (42–45, 48–50).

## 3 Hyperthermia-intrinsic effects

### 3.1 Direct cytotoxicity related to thermal shock

HT also exerts a *direct* cytotoxicity, which occurs particularly at temperatures above **≈** 42°C and increases as the temperature rises (14, 60). When a cell undergoes a thermal shock, intracellular proteins are denatured and unfolded, and end up aggregating (14, 28, 61, 62). All cellular proteins are affected: nuclear proteins malfunction, the cytoskeleton collapses and the organelles responsible for protein production (ER and Golgi) are damaged. Since nuclear proteins are fragile, a temperature of 40°C is sufficient to damage them (28). The centrosome is also affected, leading cells undergoing mitosis, particularly tumor cells which are unable to slow replication to repair damage, to die by mitotic catastrophe (62). Moreover, membrane lipids are altered, leading to increased cellular membrane permeability and mitochondrial uncoupling (28). Mitochondrial dysfunctions result in a significant release of ROS, closing the circle by indirectly bringing linking HT to DNA damage (18, 28). Through these various cellular pathways, HT appears to have the capacity to kill a portion of heated tumor cells by apoptosis (<43°C) or necrosis (>43°C), particularly in the most acidic and hypoxic areas of tumors (14, 24, 28, 63). However, the direct killing by HT seems to be significant only at temperatures above those optimizing radiosensitization (14, 24). Furthermore, protumorigenic endothelial cells and fibroblasts in the TME are particularly sensitive to thermal shock (32). Interestingly, some drugs can have a heat-sensitizing effect by modulating cell apoptosis pathways (e.g., verapamil, lidocaine), as shown *in vitro* (61), which suggests the theoretical opportunity to use thermos-sensitizers, a nice parallel to the use of radiosensitizers to enhance RT efficacy.

### 3.2 Thermotolerance

Cells actively control the integrity of their proteins: after a heat shock, unfolded and aggregated proteins result in the activation of several HSPs, through the transcription of heat-shock factor 1 (HSF1) (28, 62, 64, 65). The activation of these specific chaperones, dedicated to maintaining cellular homeostasis, result in several repair-related procedures, such as the reversal of heat-induced protein misfolding, the elimination of irreparable proteins or organelles in the lysosome (autophagy), the repair of the centrosome and cytoskeleton, the upregulation of DNA-repair proteins, the inhibition of apoptosis and the production of paracrine signals inducing heat shock response in nearby cells (28, 62, 64, 66). HSPs thus provide damaged cells with an opportunity to repair themselves before dying, if the damage is reparable (28, 62, 64, 66). Furthermore, HSP pathways are involved in the promotion of local angiogenesis, cell proliferation, adhesion, invasion and metastasis (62, 64, 66). Tumor cells exhibit numerous cellular dysfunctions and thereby possess a high basal level of HSPs activation, due to their hypoxic, acidic and nutrient-deprived microenvironment as well as their rapid division and genomic instability (64, 65, 67). Therefore, tumor cells under chronic overexpression of HSPs are not only subject to resistance to HT but also have an increased malignancy (28, 46, 62, 64–67).Following HT, a transient activation of HSPs pathways results in a temporary resistance to heat, a phenomenon known as “thermotolerance” (28, 62). Thermotolerance appears a few hours after a HT session, peaks at 24 hours, and may take up to 5 days to resolve. The exact timing and intensity of the effect depends on the cell type and the heating parameters (temperature, duration, interval between sessions, etc.): for example, the higher the temperature reached in the tumor, the longer the thermotolerance seems to last (62, 68). This has important clinical implications, as thermotolerance renders the radiosensitizing effect of HT transient, thus requiring the therapeutic interventions to be performed within an overall short time (short therapeutic window). It also requires the HT sessions to be separated by at least 48–72 hours, limiting the number of sessions that can be performed per week to one or two. Indeed, performing daily HT sessions considerably reduces its effect (28, 60, 68) and should be avoided. Alternatively, HSPs- inhibitors can be applied to reduce thermotolerance and possibly decrease tumor aggressiveness, but with the risk of a reduced heat-induced immune response (28, 46, 62, 64–67, 69).

## 4 Key mechanisms of the interaction between HT-RT

**Figure 1:**
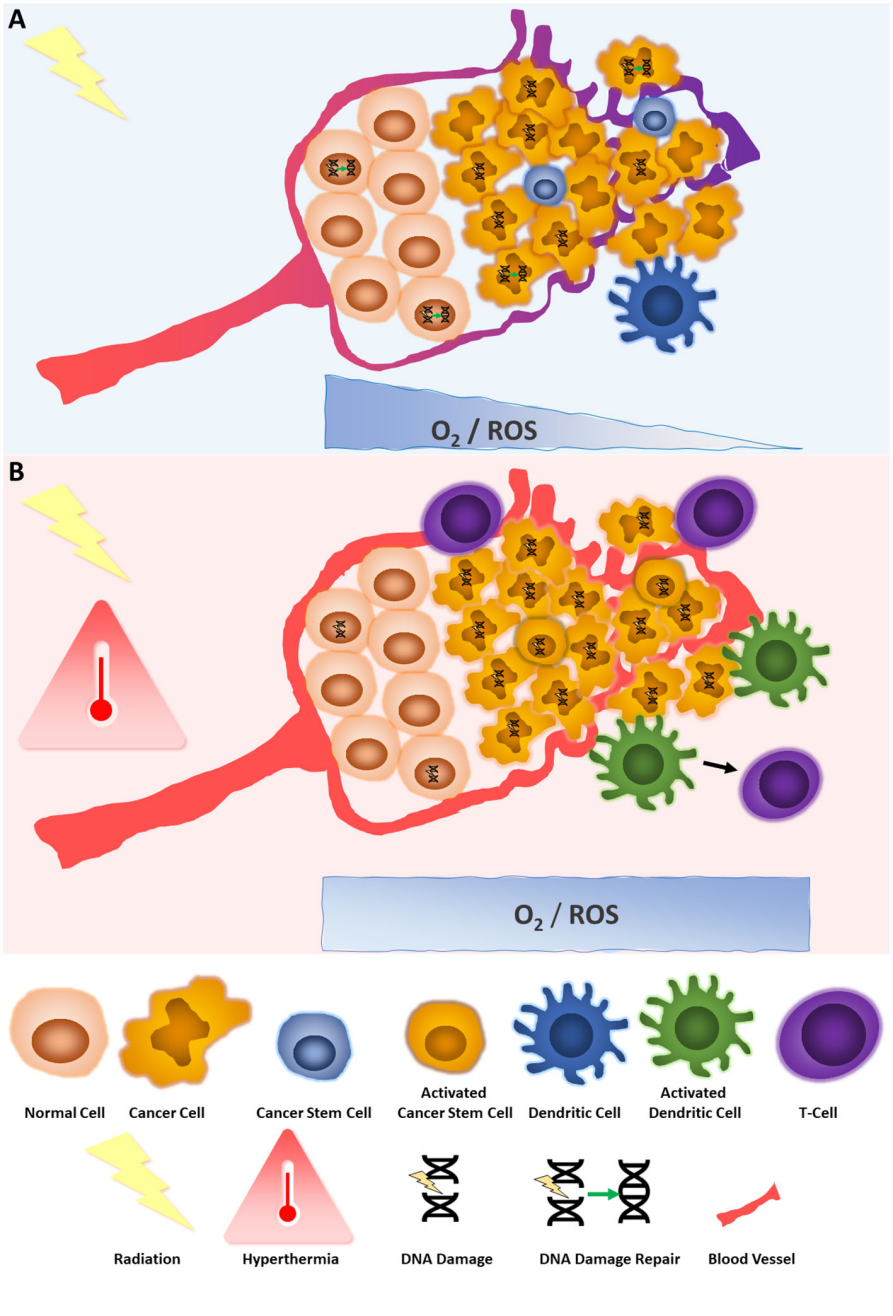
Key mechanisms of the interaction between hyperthermia and radiotherapy. **(A)** The effect of radiotherapy on tumor and normal cells. Radiation provokes DNA damage, in dividing cells, dependent on oxygen levels and therefore production of ROS. DNA breaks are repaired more readily in normal cells than cancer cells. Vessels around tumor cells are abnormal and do not supply sufficient oxygen, leading to hypoxia and less radiation-induced DNA damage. Cancer stem cells are in quiescent state and poorly affected by irradiation. Irradiation can also affect immune cells, such as dendritic cells but its immunostimulatory role is context dependent. **(B)** The effect of radiotherapy combined with hyperthermia on tumor and normal cells. Hyperthermia potentializes the effects of irradiation by reducing DNA damage repair, increasing oxygen levels by vessel dilation, increasing ROS, bringing cancer stem cells out of their quiescent state, and stimulating the immune system such as dendritic and T-cells against the tumor.

This work aimed to concisely review the underlying mechanisms of interaction between RT and HT, with the goal of bringing this synergistic effect closer to current clinical research focus. Indeed, most of the hallmarks of cancer are addressed in a beautifully complementary manner by the combination of the two modalities (**Figure 1**):

**DNA damage**: As a major radiobiologic event, it depends on ROS, leading to the temporary arrest of the cell cycle. If not quickly repaired, it will lead to cell death. Tumor cells have a reduced capacity of DNA-repair, thus are more sensitive to RT than healthy cells. HT damages several DDRs, thus enhancing RT cytotoxicity. Radioresistance is dominated by the cell cycle and the presence of DDRs: thus, TRT reverses radioresistance.

**Hypoxia:** Directly associated to radioresistance, hypoxia becomes present as tumor grows and local vasculature becomes insufficient to support its nutrient needs, leading to the development of an anarchic vascular network, reducing local oxygen partial pressure (pO_2_). HT vasodilates transitorily abnormal tumor blood vessels, inducing an increase in tumor perfusion. Therefore, the TME is reoxygenated, reversing radioresistance. However, this mechanism is only valid for temperatures <42°C.

**Stemness:** CSCs promote cancer initiation, progression, metastasis, treatment resistance and recurrence and are harbored particularly in hypoxic areas of the tumor, leading to radioresistance. HT appears to radiosensibilize CSCs, although the underlying pathophysiological processes are not understood.

**Immunostimulation:** HT possesses only immunostimulatory effects, through inflammation, mediated by HSPs, leading to increased immunogenicity, leukocyte infiltration, vasodilation and ultimately ICD. RT produces both immunostimulatory and immunosuppressive effects: by releasing TAAs, stimulating inflammatory cytokines, promoting dendritic cells maturation and preparing antigen presentation to T and NK cells by the immune host system, it makes the tumor an *in situ* vaccine, while, by damaging leukocytes situated in the tumor, stimulating other anti-inflammatory cytokines and attracting CAFs/TAMs/MDSCs/Treg, it can create a radioresistant state in tumors and upregulate PD-L1. The RT-induced abscopal effect is an immunogenic effect that could potentially be enhanced with TRT.

Two HT-intrinsic mechanisms of action, which represent compensatory responses of cell to heat, are also described: direct tumor kill related to thermal shock, present only at higher temperatures, can further contribute to HT cytotoxicity; thermotolerance, on the other hand, represents a limitation of HT for anticancer treatments.

## 5 Challenges, perspectives, and conclusion

Several physiological mechanisms explaining the synergy of TRT are now understood. Nevertheless, other gray zones remain, such as the optimal temperature for achieving the most effective radiosensitizing effect, the duration of heat-induced vasodilation (14, 24), the pathophysiology underlying CSCs and quiescent cells radiosensitization by HT (32), the exact molecular mechanisms underlying the DNA repair inhibition (18, 19) and the optimal parameters to obtain clinically meaningful benefits from the TRT-induced immunostimulation (42, 43) (22, 48–50).

Several strategies to enhance both the local and systemic effects of TRT in tumor kill are attempted or provide interesting opportunities to be explored: the addition of systemic therapies, such as ICIs (43–45), drugs increasing local oxygen availability (11, 27, 30), vascular-disrupting agents (VDAs) (14, 70), DNA-repair inhibitors (16, 18, 19), HSP inhibitors (28, 62, 69), antioxidant enzyme inhibitors (12, 71) or heat-sensitizing molecules (61) could theoretically increase the synergistic effect. This, however, remains to be seen: the paradigm of radiation sensitizers and radioprotectors and the challenges of its clinical implementation has shown that these combinations can be challenging in terms of selectivity and practical implementation (72).

Both the technological evolution as well as the improved understanding at a mechanistic level, of both RT and HT, offers exciting opportunities: high-precision radiotherapy, resulting in oncological efficacy and remarkably improved treatment tolerance, in the field of RT, improved heat delivery planning and thermometry (e.g., MRI thermometry), accumulation of improved quality clinical data, in the field of HT, promise new therapeutic opportunities for modern TRT.

An increasing number of preclinical and clinical studies are focusing on heating tumors with nanoparticles: magnetic nanoparticles (MNPs) generate heat when exposed to an alternating magnetic field, while gold nanoparticles are excited by light (73). Injected intravenously, nanoparticles have the advantage to preferentially accumulate in tumor tissues due to the abnormal permeability of their immature neo-vessels (Enhanced Permeability and Retention effect). Absorbed by tumor cells through endocytosis, nanoparticles can also directly cause various cellular dysfunctions, such as oxidative stress, cytoskeleton disruption or DNA damage (73, 74). Since nanoparticles hold great promise for heating tumors more precisely and selectively, they are now being clinically tested in association with RT for the treatment of glioblastoma and prostate cancer, and are in preclinical stages for other indications (75, 76). Although a lot needs to be undertaken before these advancements become ready for prime-time, they promise high-precision TRT and could permit to refine the already established synergy between RT and HT. These perspectives hold promise for cancer cure and merit study with established modern tools, incorporating translational aspects that will then be brought to the patient.

## Author contributions

MR: Conceptualization, Investigation, Methodology, Visualization, Writing – original draft, Writing – review & editing. AD: Conceptualization, Methodology, Project administration, Supervision, Visualization, Writing – review & editing. PT: Conceptualization, Methodology, Project administration, Supervision, Visualization, Writing – review & editing.
